# Neuroprotective Effect of Coumarin Nasal Formulation: Kindling Model Assessment of Epilepsy

**DOI:** 10.3389/fphar.2018.00992

**Published:** 2018-09-03

**Authors:** Suraj Muke, Aakruti Kaikini, Vaibhavi Peshattiwar, Sneha Bagle, Vikas Dighe, Sadhana Sathaye

**Affiliations:** ^1^Department of Pharmaceutical Sciences and Technology, Institute of Chemical Technology, Mumbai, India; ^2^Preclinical Reproductive and Genetic Toxicology, National Institute for Research in Reproductive Health, Mumbai, India

**Keywords:** coumarin fraction, coumarin nasal formulation, epilepsy, kindling, pentylenetetrazole, neuroinflammatory marker

## Abstract

Epilepsy is a brain disorder characterized by sudden recurrent seizures. Considering the fact that epileptogenesis is a process that affects the quality of life, our goal is to delay the process of epileptogenesis and to increase the latency of epileptic attacks, offering better quality of life to patients. Traditional system of medicines has a promise in some of the medicines, which have been used for the treatment of epilepsy. One such medicinal plant is *Eclipta alba* (EA). According to Ayurvedic philosophy, the juice of leaves of EA is pounded with garlic and pepper for the treatment of epilepsy. Taking clue from the Ayurvedic system of medicines, we formulated coumarin fraction of EA, namely, coumarin nasal formulation (CNF) for its nasal delivery. CNF was analyzed by using high performance liquid chromatography (HPLC) and ultraviolet absorption spectroscopy for its drug content determination. *In vitro* drug release studies were performed in simulated nasal electrolyte solution (SNES) maintaining constant pH of 5.5 at 37°C. Irritation by CNF was evaluated using hen’s egg test chorioallantoic membrane (HET-CAM) assay. Formulation was found to be non-irritant in HET-CAM assay. CNF was further assessed *in vivo* by measuring the progress and attainment of pentylenetetrazole (PTZ) kindling in mice. Neuronal changes were assessed by hematoxylin and eosin (H&E) and Nissl staining technique. Glial fibrillary acidic protein (GFAP) a neuroinflammatory marker and tumor necrosis factor alpha (TNF-α) an inflammatory marker were also measured. CNF (10 mg/kg, nasal route) when given as a pretreatment lowered seizure score and delayed the progression of seizure similar to diazepam. CNF decreased the PTZ induced oxidative damage, TNF-α as well as GFAP levels in the midbrain tissue particularly in hippocampus region. The results suggest that CNF may be a promising therapeutic approach to offer protection from sudden recurrent seizures alone or in combination with current drugs in management of epilepsy.

## Introduction

Epilepsy is a neurological disorder characterized by transient occurrence of abnormal excessive synchronous neuronal firing in the brain. The predominant cognitive, psychological, and behavioral manifestations in epilepsy further aggravate the vulnerability of seizures incidence, which eventually leads to plaintive quality of life of patients suffering from it (Chang and Lowenstein, 2003). According to World Health Organization, the proportion of general population with active epilepsy at a given time is about 4 to 10 per 1000 people. The epilepsy scenario is far more alarming in the developing nations with a proportion of about 7–14 per 1000. This disorder has been found to prevail since ages and presently affects approximately 50 million population worldwide (Epilepsy, 2012). The signs and symptoms include warnings, such as visual or sensory auras, tingling fingers, altered awareness, and convulsive or abnormal movements. The pathophysiology underlying the epileptic process includes mechanisms involved in instigation of seizures (ictogenesis), in addition to those involved in transforming the normal brain into a seizure-prone brain (epileptogenesis) (Fisher et al., 2005). Currently, no antiepileptogenic treatments are available in the market that prevents the progression of epileptogenesis. The epilepsy plight suggests the imperative need for the development of novel therapeutic strategy. The rational approach in designing the new strategy should consider molecules which block both ictogenesis and retard the progression of epileptogenesis. The ideal anticonvulsant drug should constrain seizures resulting from the rapid and excessive firing of the neurons. Additionally, the drug therapy currently available as antiepileptic drugs (AEDs) is associated with side effects and dose related chronic toxicity involving vital organ system. Furthermore, all the currently available AED have potential for adverse effects like cognitive and behavioral impairment. The cost of new AED, namely, oxcarbamazepine, lamotrigine, felbamate, gabapentin, and vigabatrin is three to six times higher than the conventional AEDs like phenytoin, carbamazepine, and valproic acid which is a major concern and important factor for a physician to prescribe (Britton and So, 1996). In Ayurveda system of medicine, EA is considered a *rasayana* for longevity and rejuvenation. The leaves of EA are used to treat epilepsy in India (Pakrashi and Chatarji, 1994). EA has been used as a traditional healer and medicine especially in the southern regions of India for the treatment of epilepsy since ancient times (Reddy et al., 1989). EA extract showed significant decrease in the locomotor activity at the dose of 50 mg/kg and dose dependent protection from seizures in maximal electroshock model (Bikjdaouene et al., 2003). Above study confirmed the antiepileptic potential of the extract possibly due to presence of phytoconstituents like wedelolactone, luteolin, and β-amyrin in it (Shaikh et al., 2012). There are studies indicating that EA phytoconstituents also have affinity toward benzodiazepine (BZD) binding site on the GABA receptor (Coleta et al., 2008). EA contains wide range of active phytoconstituents including coumestans, alkaloids, flavonoids, glycosides, sterols, and triterpenoids. These EA fractions were screened in various models of acute and chronic epilepsy (unpublished data from our lab). In preliminary study, we found that coumarin fraction (100 mg/kg) exhibited an excellent antiepileptic activity in acute PTZ induced seizure model in mice. Considering these literature reports and results, we designed the present study to formulate the active fraction for its anti epileptic potential in kindling model of epilepsy.

## Materials and Methods

### Plant Material

The leaves of the EA plant were procured from the local market in Mumbai. Professor Ganesh Iyer, Department of Botany, Ruia College, Mumbai, India, authenticated the specimen. The voucher specimen (No. SSS/1313/15) of the same was deposited at the Institute of Chemical Technology for future reference.

### Drugs and Chemicals

Pentylenetetrazole (Sigma Chemical Co., St. Louis, MO, United States), diazepam (Mumbai, Roche Pharmaceuticals, India), and wedelolactone (Natural remedies Private Limited, Bangalore, India) were used in the present study. The drugs were dissolved in distilled water and subsequently used for administration. The solvents used for the extraction in the study were, petroleum ether, methanol, diethyl ether, and ethyl acetate (S.D. Fine-Chem, Mumbai, India). The surfactants like Tween 80, polyethylene glycol 300, benzalkonium chloride, citric acid, and sodium citrate (S.D. Fine-Chem, Mumbai, India) were used in formulation. All other chemicals and reagents used in the experiments were of analytical grade.

### Preparation of the Extracts

The leaves of EA were dried in shade and stored at 30°C temperature. They were crushed further to obtain a coarse powder and passed through a sieve (no. 40). Shade dried and powdered leaves (500 g) were subjected to soxhlet extraction with petroleum ether and methanol (60–80°C) for 24 h. After completion of the extraction, the solvent was removed by distillation and the extract was concentrated under vacuum (40°C) to yield the petroleum ether extract of EA and methanolic extract of EA, respectively. Dried greenish crystals of methanolic extract were further partitioned with solvent diethyl ether to get coumarins from EA.

#### Isolation of Coumarins From EA

The methanolic extract of EA (30 g) soaked in distilled water containing 2% acetic acid (300 ml) and heated for 1 h. The extract was filtered using Whatman No. 42 paper. Filtrate was then evaporated to 100 ml under reduced pressure at a temperature of 40°C. Filtrate (100 ml) was added to the same amount of solvent diethyl ether. The whole (200 ml) was then partitioned using separating funnel by standing for 30 min at room temperature after vigorous shaking, which was repeated five times until diethyl ether fraction was no longer fluorescent under long UV light (366 nm). Diethyl ether was evaporated to get concentrated solution (100 ml). On cooling at 0–4°C, a greenish yellow precipitate formed at the bottom of the flask was separated. It was dried and weighed (Prajapati and Patel, 2012). Greenish yellow dried powder of coumarin fraction was stored in fridge until further use.

### Preparation of the Coumarin Nasal Formulation and Stability Studies

The formulation comprising of coumarin fraction, isolated and characterized, was formulated in solution form and denoted as CNF [1% coumarin fraction (w/v) total solids]. CNF comprised of 1gm of coumarin extract per 100 ml of formulation comprising Tween 80 (1% v/v) + polyethylene glycol 300 (1.5% v/v) + benzalkonium chloride (0.1% v/v) + 0.1 M citrate buffer pH 7.4 (1.05 g citric acid and 1.47 g sodium citrate in water for injection) to make up the volume to 100 ml. CNF was stored in an amber color vials until further analysis. Stability studies were carried out as per the ICH guidelines at 25 ± 2°C/60 ± 5% RH and 2–8°C. The average particle size (if turbidity occurred) and drug content were evaluated at 1, 2, and 3 months.

#### Phytoconstituents Analysis of Coumarin Fraction and CNF by HPLC

A HPLC system of JASCO Corporation was used for the analysis. HPLC was equipped with auto sampler and PDA detector system. The chromatogram was analyzed by JASCO ChromNAV version 1.19.01 software. In house method was developed using stationary phase C18 column (4.6 mm × 250 mm; 5 μm) by Jasco corporation with mobile phase composition of acetonitrile and 0.1% formic acid and buffer pH adjusted to 2.5 with triethylamine, operated at 1 ml/min flow rate. Ethyl acetate was used for preparation of sample preparation. Dried powder of coumarin fraction as in 2.3.1 was dissolved in methanol (1.0 mg/mL), filtered by 0.22 μm filter, and directly injected into HPLC for the analysis of major phytoconstituents. Jasco Corporation HPLC system was used with auto sampler and PDA detector for the analysis. Wedelolactone, luteolin, and apigenin were separated and identified on Thermo BDS Hypersil-C18 (250 × 4.6, 5 μ) with Purospher^®^ STAR RP-18e (5 μm) guard column. Wedelolactone was eluted isocratically and detected on UV PDA detector at wavelength of 352 nm. Quantitative estimation of wedelolactone in coumarin fraction and CNF was determined. Luteolin and apigenin were eluted isocratically with 50% acetonitrile and 50% formic acid buffer (0.1%, pH 2.5) at flow rate of 1.0 ml/min and detected at wavelength of 325 nm. Chromatogram for wedelolactone, apigenin, and luteolin in coumarin fraction are shown in **Figure 2**.

#### Wedelolactone Content of CNF

One milligram of CNF was diluted in 100 μl methanol and 900 μl PBS at pH 7.4 under continuous stirring for 10 min and further subjected to centrifugation at 2000 rpm for 10 min (Remi Centrifuge CPR 24 plus Centrifuge). Wedelolacone (WL) content was quantified in the supernatant by UV spectroscopy at 350 nm (UV Epoch microplate reader Biotek). Calibration curve of WL in PBS was linear (*R*^2^ = 0.999) in the range of 10–50 μg/ml. All experiments were repeated in triplicate. Drug content was determined according to the following equation.

% drug Content=Weight of WL in Formulation (mg)/Weight of Formulation (mg)×100

#### Solubility Study of CNF

Solubility of coumarin extract, containing wedelolactone, was determined in various surfactants (Labrasol, Brij 58, Solutol HS 15, Tween 20, Tween 80) and co-surfactants (PEG 300, PEG 400, PEG 600, Transcutol P, and Glycerol). Briefly, excess coumarin extract was added to 1 ml surfactant or co-surfactant separately in eppendorf tubes. The tubes were sealed and vortex mixed intermittently followed by sonication and equilibration at room temperature for 24 h. Equilibrated samples were centrifuged at 20,000 rpm for 10 min. Aliquot of 0.1 ml was withdrawn from the supernatant and diluted appropriately with methanol. Diluted sample was analyzed for wedelolactone by UV spectrophotometry at 354 nm. All experiments were performed in triplicate.

#### Fourier Transform-Infrared (FTIR) Study

Fourier Transform-Infrared spectra of the crude fraction and formulation were recorded to confirm the functional groups and structural similarity between coumarin fraction and CNF. FTIR was recorded using an IR Prestige-21 spectrometer (Shimadzu, Kyoto, Japan) in the range between 4000 and 500 cm^-^1 with a resolution of 1 cm^-1^ after 57 scans.

### *In vitro* Release Studies

*In vitro* drug release studies for CNF and coumarin fraction for the determination of wedelolactone release were carried out in SNES (NaCl 7.45 g/L, Kcl 1.29 g/L, and Cacl2 0.32mg/L) pH 7.4 at 35 ± 0.2 °C. Dialysis bag method was used [dialysis membrane (HIMEDIA LA 395-5MT), avg. flat width 32.34 mm and avg. diameter 21.5 mm] in a beaker loaded with magnetic stirrer. Dialysis bags (previously soaked overnight in distilled water) were filled with 10 mg of formulation dispersed in 1 ml of phosphate buffer solution (PBS) pH 7.4 preheated at 35°C. Dialysis bags were then sank in small screw-top glass vials filled with 20 ml of SNES solution (35°C), sealed with Parafilm^®^ and placed in the water bath under mild agitation. At predetermined time intervals (0, 5, 15, 30, 60, 120 min, 12 h, and 24 h), 200 μl samples were withdrawn and replaced with equal volumes of fresh and preheated SNES solution. Quantification of WL in the samples was performed spectrophotometrically at 350 nm (UV Epoch microplate reader Biotek). *In vitro* release experiments were repeated in triplicate.

### *Ex vivo* Permeation Studies of CNF on Goat Nasal Mucosa

*Ex vivo* permeation study were carried out across goat nasal mucosa collected from the local (Deonar, Mumbai) slaughter house. Immediately after the animal sacrifice, the right and left nasal concha were isolated and nasal epithelium was carefully excised, cleansed, and immersed in icecoldphosphate buffer pH 7.4. The nasal epithelium (mucosal thickness ca. 100 μm) was immediately mounted on a jacketed vertical Franz cell (4.91 cm^2^ active surface area and receptor volume: 20 ml) under mild magnetic stirring (20 rpm), with the mucosal surface facing the donor compartment and the serosal side facing the receptor compartment. The membrane integrity and proper arrangement on the cell surface was assessed carefully. Tissue thermal equilibrium was achieved by filling both compartments with prewarmed (35 ± 0.2°C) SNES solution (pH 5.5). After 20 min, buffer solution was removed from the donor chamber and replaced by a dispersionof 1 ml of 1 mg/ml of CNF formulation, solution of coumarin fraction of the same concentration in PBS was used as control. The donor compartment was sealed with Parafilm^®^ throughout the experimental procedure. At predetermined time intervals (15, 30, 60, 120, 240, 360, and 480 min), samples of 0.5 ml were withdrawn from the receptor compartment and replaced with equal volume of preheated SNES solution. All experiments were repeated at least three times. Samples were centrifuged at 10,000 rpm for 15 min (Eppendorf centrifuge Remi Centrifuge CPR 24 plus) and the supernatants (60 μl) were directly loaded into HPLC vials to quantify WL content, under the following chromatographic conditions: HPLC setup comprised of a PU-2089 plus quaternary gradient pump, AS-2055 plus intelligent auto sampler, and a MD-2018 plus photodiode array detector (JASCO). The HPLC column used was Thermo Scientific syncronis C18, 150 mm × 4.6 mm, particle size 5 μm. Mobile phase consisted of ACN: 0.1% formic acid 60:40 (v/v), 10 mM, pH 5.5 (pH adjustment with triethylamine 1 M). Flow rate was regulated at 1 ml/min; injection volume at 50 μl. Retention time was approximately three minutes at ambient temperature conditions. Calibration curve was linear (*R*^2^ = 0.997) over the concentration range of 10–1000 μg/ml. Steady state flux (Jss) is determined by calculating the slope of the linear portion of the curve obtained after plotting the cumulative amount of drug permeating nasal epithelium per unit area (μg/cm^2^) against time (Fisher et al., 2005). Consequently, apparent permeability coefficient, P (in cm/s) is calculated as follows:

$$P=\text{Jss/Cd, where Cd is the initial concentration in the donor compartment}(\mu g/{cm}^{3}).$$

Subsequently, for both CNF and coumarin fraction, the apparent permeability coefficient was calculated and compared on the basis of enhancement ratio (*R*).

$$R=P_{\left(CNF\right)}/P_{\left(\text{Coumarin Fraction}\right)}$$

Steady state flux (Jss) was calculated according to method developed by Karavasili et al. (2016).

### Evaluation of CNF Irritancy Potential Using Hen’s Egg Test Chorioallantoic Membrane (HET-CAM)

The HET-CAM assay was performed following “The Interagency Coordinating Committee on the Validation of Alternative Method” recommendations. Some modification in method were adopted as per (Dehelean et al., 2011) to suit our laboratory conditions. The purpose of this protocol was to describe the components and procedures used to assess the potential nasal irritancy of a formulation as measured by its ability to induce toxicity in the chorioallantoic membrane of a chicken. Fertilized eggs were procured from Central Poultry Development Organization (Western Region) Mumbai. Study was divided into four groups each group containing six eggs, group I: normal control (saline 0.9%), group II: vehicle control [Tween 80 (1% v/v), polyethylene glycol 300 (1.5% v/v), benzalkonium chloride (0.1% v/v) in 0.1 M citrate buffer pH 7.4 (1.05 g citric acid and 1.47 g sodium citrate in water for injection)], group III: negative control (1% sodium dodecyl sulfate in 1 M potassium hydroxide solution), group IV: CNF. Parameters observed were the onset of (1) hemorrhage; (2) coagulation; and (3) vessel lysis. These results were considered individually and then combined to derive irritancy score (Fisher et al., 2005), which is used to classify the irritancy level of the formulation. Results were analyzed by using method described by some modification to available literature (Kishore et al., 2008) with reference to irritation severity score. After the treatment time of 5 min of a particular component or formulation, irritation severity was determined by either hemorrhage or lysis, or coagulation. The following scheme: 0–1 = non-irritant; 1–5 = slight reaction; 5–9 = moderate reaction; 9–21 = severe reaction. Mean scores were determined further (Luepke, 1985; Kalweit et al., 1987, 1990).

### *In vivo* Evaluation of Coumarin Fraction and CNF

#### Experimental Animals: Rodents

Swiss male albino mice (20–25 g) were procured from Bombay Veterinary College, Parel, Mumbai, India, and were acclimatized in the animal house of the Institute of Chemical Technology (ICT), Matunga, Mumbai. Young healthy male mice were housed eight per cage and maintained at a temperature of 23 ± 2°C, at a humidity of 51 ± 10% and in a 12:12-h light/dark cycle with free access to rodent chow by Pranava Agro Industries Ltd., Sangli, India, and tap water *ad libitum*. The experiments were carried out between 9 AM and 6 PM. The animal study was approved by the Institutional Animal Ethics Committee (ICT/IAEC/2017/P30), Mumbai.

#### Evaluation of Coumarin Fraction in Acute PTZ-Induced Seizure in Mice

Male albino mice with a body weight between 18 and 22 g comprising of six mice in each experimental group were used. Experimental groups taken were Group I: normal control (saline only), Group II: positive control (diazepam 2 mg/kg i.p. dose), Group III: negative control (PTZ 100 mg/kg i.p. dose), Group IV: treatment (PTZ+ coumarin fraction 50 mg/kg i.p. dose), Group V: treatment (PTZ+ coumarin fraction 75 mg/kg i.p. dose), and Group VI: treatment (PTZ+ coumarin fraction 100 mg/kg i.p. dose). Pre-treatment was given 30 min prior to i.p. injection PTZ 100 mg/kg. Each animal was placed into an individual plastic cage for observation lasting 30 min. Seizures and tonic–clonic convulsions were recorded and score was assigned as follows, onset of myclonic jerks; onset of clonic seizures; onset of tonic seizures; status of animal (recovery or death; Smith et al., 2007).

#### Pharmacokinetic and Brain Distribution of CNF

Male wistar rats (240–270 g) were initially subjected to the jugular vein cannulation using polyethylene cannula tubing (0.5 mm ID × 1.0 mm OD). The rats were anesthetized using ketamine (40 mg/kg) and xylazine (4 mg/kg) intraperitoneally (*i.p.*) and the jugular vein was cannulated for the purpose of blood collection. The entire PK study was broadly divided into four groups, Group I: single intravenous bolus dose of wedelolactone (0.4 mg/kg i.v.), Group II: CNF (0.4 mg/kg i.v.), Group III: CNF (0.4 mg/kg nasal), and Group IV: CNF (0.4 mg/kg i.p). Serial blood samples (0.250 ml) were collected via the jugular vein at 0.083, 0.25, 0.5, 1, 2, 4, 8, 12, and 24 h after wedelolactone and CNF administration. At each time point, the amount of withdrawn blood volume was replaced by heparinized saline via the jugular cannula (Mandlekar et al., 2007). The plasma samples were obtained after centrifugation of blood samples at 10,000 × *g* and stored at -80°C until HPLC analysis. Brains of rats were isolated at 12 and 24 h (*n* = 4) for brain distribution study. Brain was quickly removed and rinsed with 0.9% saline. The brain tissues were homogenized using ice-cold 0.1 M PBS (pH 7.4). The homogenate was centrifuged at 4°C (3000 rpm; R-248M of CPR-223 24 plus Instrument, Remi, India) for 15 min. The aliquots obtained were used for the estimation of wedelolactone content (Omura et al., 1965; Walawalkar et al., 2006). Linearity for wedelolactone was done using reaction mixture (300 μl) consisting of 0.1 M sodium phosphate buffer pH 7.4, plasma matrix or brain homogenate, and wedelolactone (0, 1, 3, 10, 30, and 100 μg/ml). All reactions were carried out in duplicate and mean value obtained from each set of duplicate incubations were used for further calculations.

#### Evaluation of CNF in PTZ Induced Kindling Model of Epilepsy

**FIGURE 1:**
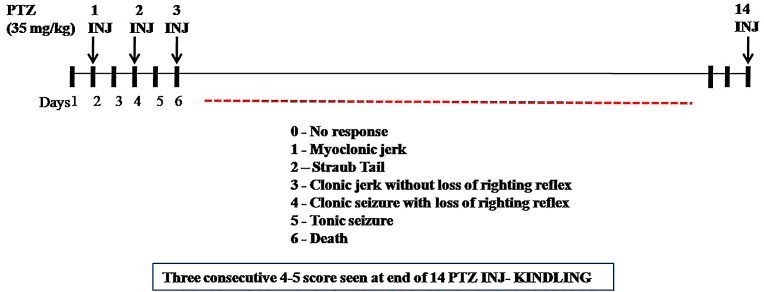
Protocol for PTZ induced kindling.

According to Tambe et al. (2016), repetitive administration of PTZ (35 mg/kg, i.p.) as a subconvulsant dose on every alternate day (Monday, Wednesday, and Friday) induces kindling in mice. After each PTZ injection, the convulsive behavior was observed for 30 min. The intensity of seizure response was scored according to (Shaikh et al., 2013): Score 0 (no response); Score 1 (myclonic jerk); Score 2 (straub tail); Score 3 (clonic jerk without loss of righting reflex); Score 4 (clonic seizure with loss of righting reflex); Score 5 (tonic seizure), and Score 6 (death). The maximum seizure response was recorded in each animal. Kindling occurs when animal gets Stage 4 or Stage 5 for consecutive 3 days after PTZ administration (**Figure 1**). The experimental groups taken were as follows, Group I: positive control (diazepam 1 mg/kg i.p. dose), Group II: negative control (PTZ 35 mg/kg i.p. dose), Group III: normal control (saline only), Group IV: vehicle control (PTZ + vehicle for nasal dose), Group V: treatment (PTZ+ CNF 5 mg/kg nasal dose), and Group VI: treatment (PTZ+ CNF 10 mg/kg nasal dose).

##### Evaluation of superoxide dismutase, catalase, reduced glutathione, and malondialdehyde levels

All groups were sacrificed at the end of the study; brain was quickly removed and rinsed with 0.9% saline. The brain tissues were homogenized using ice-cold 0.1 M PBS (pH 7.4). The homogenate was centrifuged at 4°C (3000 rpm; R-248M of CPR-223 24 plus Instrument, Remi, India) for 15 min. The aliquots obtained were used for the estimation of SOD, CAT, GSH, and malondialdehyde (MDA) using methods described by Sinha (1972); Nandi and Chatterjee (1988), Ellman (1959), and Ohkawa et al. (1979), respectively.

##### Histopathology

After fixation with 4% paraformaldehyde, the brain samples were routinely processed and subjected to paraffin embedding. The coronal sections of 10 μm passing through hippocampus were sliced, mounted and stained by H&E and observed under microscopes at different magnifications. The sections were assessed for the alterations pertaining to neuronal damage like pyknotic nuclei, distorted morphology of cell, etc.

##### Immunohistochemistry

Immunohistochemistry was performed according to (Tambe et al., 2016)on a previously fixed, 5-μm-thick brain sections (consisting 10–12 sections) passing through hippocampus. Sections were dried and fixed on poly-L-lysine coated glass slides at 37°C for 24 h. Xylene was used for de-waxing. The sections were rehydrated with decreasing concentrations of alcohol, namely, 95, 70, 50% for 5 min in each concentration. Peroxidase activity was blocked by incubating with 3% hydrogen peroxide in methanol for 30 min. Sections were washed with distilled water for 5 min. Antigen recovery was initiated by heat mediated antigen recovery technique using citrate buffer pH 6 for 15 min. followed by washing with distilled water for several times. Subsequently, it was treated with PBST (0.3% Triton X-100) for creating pores in sections to absorb antibody. Primary monoclonal anti-GFAP antibody (ab10062) was added and incubated for overnight at 4°C. After washing with PBS, secondary antibody Alexa fluor @ 488 (goat anti-mouse) was added to it and incubated for 1.5 h. After giving a secondary wash in PBS, it was incubated with DAPI (1.5% in PBS). Number of positive cells to GFAP in the slides were counted under a light microscope at a intensification of 40x (Matsuda et al., 2009).

##### Determination of TNF-α by ELISA

Tumor necrosis factor alpha, a pro-inflammatory cytokine, plays a major role in initiating and regulating the cytokine cascade during an inflammatory response. Brain homogenates prepared were used for the determination of TNF-α. ELISA plate was incubated overnight with Capture antibody in coating buffer at 4°C. After washing with the wash buffer, the wells were blocked with assay diluents and incubated at room temperature for an hour. Standard curve was plotted against rat standard TNF-α (eBioscience, United States). Subsequently, the plate was incubated with the samples for 2 h at 4°C. Brain TNF-α was estimated by using ELISA kit (eBioscience, United States) as per the methodology provided by manufacturer in the kit. Finally, readings were taken at 450 and 570 nm and analyzed.

### Statistical Analysis

Data of all the results were presented as mean ± SEM. The analyses of all the studies were done with the help of ANOVA followed by Dunnett’s test. The difference in the results of PTZ and MES were considered statistically significant when ^∗^*P* < 0.05, ^∗∗^*P* < 0.01, and ^∗∗∗^*P* < 0.001 compared to negative control.

For *in vitro* and *ex vivo* permeation study, statistics were performed by paired *t*-test. Differences were considered statistically significant at *P* < 0.05.

## Results

### Extraction of Different Phytochemical Fractions From EA

The coumarin fraction was successfully separated from methanolic extract of EA with % yield of 7.6% w/w.

### HPLC Determination of Coumarin Fraction and CNF

**FIGURE 2:**
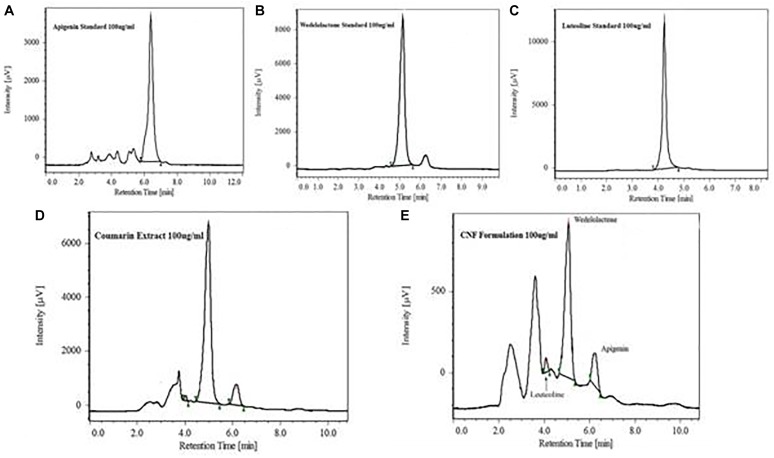
HPLC chromatogram showing major peak of standard apigenin **(A)**, standard wedelolactone **(B)**, standard luteolin **(C)**, wedelolactone in coumarin fraction **(D)**, and CNF **(E)**.

Coumarin fraction and CNF both revealed similar phytoconstituents, i.e., wedelolactone, apigenin, and luteolin with different mobile phase combination. As evident from **Table 1**, wedelolactone as a major component was eluted isocratically with 45% acetonitrile and 55% formic acid buffer (0.1%, pH-2.5) at flow rate of 1.0 ml/min and detected on UV PDA detector at wavelength of 352 nm; 81.18 and 10.97% of wedelolactone is present in coumarin fraction and CNF, respectively. Quantitative estimation was calculated on the basis of peak area with reference to the respective standards. Luteolin and apigenin present in the coumarin fraction and CNF were isocratically eluted with 50% acetonitrile and 50% formic acid buffer (0.1%, pH 2.5) at flow rate of 1.0 ml/min and detected at wavelength of 325 nm (**Figure 2**).

**Table 1:** HPLC analysis of coumarin fraction and CNF.

Compound name	Drug % in coumarin fraction	Drug % in CNF
Wedelolactone	81.18	10.97
Apigenin	4.37	0.95
Luteolin	1.24	0.56


### CNF Stability Studies

Formulated CNF was slightly yellowish in color with pleasant odor. CNF shows turbidity after 3 months at normal storage condition so the particle size was carried out by multimodal size distribution on a Nanobrook^®^. CNF was diluted with water with 1: 10 ratio (v/v). The effective diameter found was 953.81 nm with poly-dispersity index of 0.404 which indicates that the formulation (CNF) should be stored in refrigerated condition.

#### Drug Content Determination of CNF

Coumarin nasal formulation revealed high values of drug content. On the basis of linearity of WL, the CNF was reported to contain 1.70% w/v of WL and recovery yield was 34.79641% of WL as evident from **Table 2**.

**Table 2:** Drug content over accelerated stability of CNF.

Formulation	Drug content % (w/v)	Yield %
CNF (1 mg/ml)	0.98 ± 0.02	100
CNF (1 mg/ml) at 2 months	0.97 ± 0.03	98.83
CNF (1 mg/ml) at 3 months	0.96 ± 0.03	98.07


*Results are the mean values ± SD (*n* = 3) at all interval of the study (0, 2, and 3 months).*

#### Solubility Study of CNF

**FIGURE 3:**
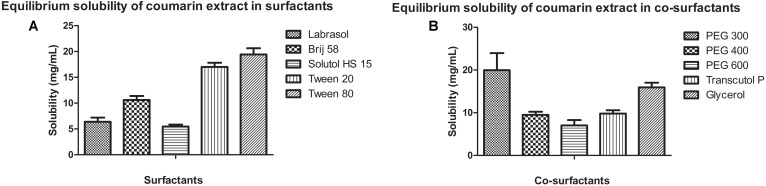
Equilibrium solubility of coumarin extract in **(A)** surfactants and **(B)** co-surfactants.

**Figure 3** shows the equilibrium solubility of coumarin extract in various surfactants and co-surfactants. Coumarin extract exhibited maximum solubility in Tween 80 and PEG 300. Thus, Tween 80 was chosen as the surfactant and PEG 300 as the co-surfactant.

#### FTIR Analysis

**FIGURE 4:**
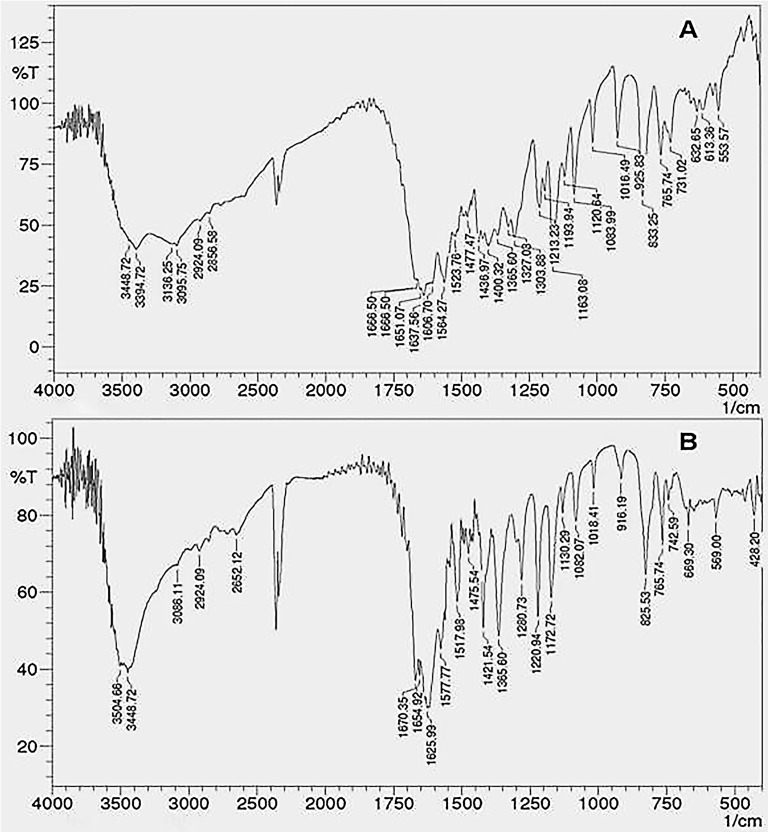
Fourier transform-infrared of **(A)** coumarin fraction and **(B)** CNF.

Fourier transform-infrared spectra of the coumarin fraction and CNF are as shown in **Figure 4**. The FTIR spectrum exhibited peaks at 3448 cm^-1^due the OH groups and a strong band at 2924 cm^-1^ (aliphatic C–H stretching) indicating the presence of –CH2– and –C–H in addition to peaks at 1606, 1523, and 1477 cm^-1^ which indicated the presence of an aromatic ring system. Broad and strong peak in the range of 1665–1685 cm^-1^ confirms the presence of α,β-unsaturated ketone or aromatic ketone. Several medium-weak multiple bands ranging from 1400 to 1600 cm^-1^ indicate the presence of C = C bending in aromatic ring while the strong peaks belonging to 1000–1300 cm^1^ are the identities of cyclic ethers. Strong, broad peaks within 3200–3600 cm^-1^ are due to phenolic OH– groups.

### *In vitro* Release Studies

**FIGURE 5:**
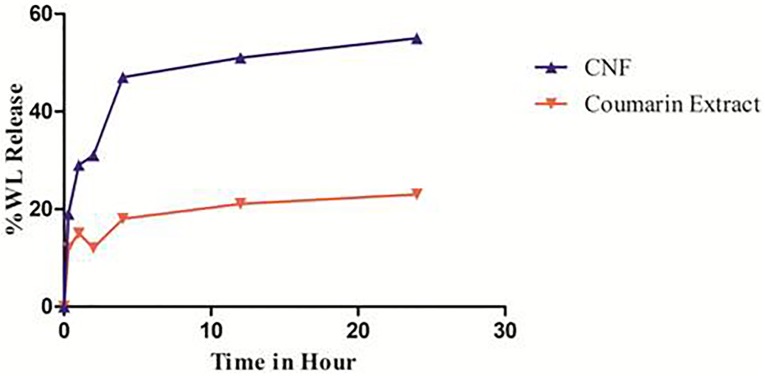
*In vitro* release pattern of CNF and coumarin fraction. Data presented as mean values ± SD (*n* ≥ 3) by paired *t*-test. Differences were considered statistically significant at *p* < 0.05.

*In vitro* release of WL from CNF and coumarin fraction was assessed in SNES solution prepared in PBS (pH 7.4) at 37 ± 0.2°C for a time period of 24 h. Cumulative drug release profiles are illustrated in **Figure 5**. Calibration curve of wedelolactone in SNES solution revealed linearity (*R*^2^ = 0.999) in the concentration range of 10–50 μg/ml.

Coumarin nasal formulation release patterns achieved a total 19% WL release within the first 30 min. Thereafter, it followed a slower release profile, reaching to a total drug release of 55.04% over the time period of 24 h, contrary to coumarin fraction which show 12% of total release of WL within first 30 min and total drug release of 23.05% over the time period of 24 h.

% Cumulative release of WL is plotted against time (h) and the slope of the linear section of the curve is calculated. Consequently, the enhanced release is calculated by taking the ratio of the two slope values. The enhanced release ratio is found to be 1.62. Thus, CNF releases 1.62 times more WL than the coumarin fraction over total period of time. In first 15 min, CNF released 1.59 times more WL compared to the coumarin fraction. This may help enhancement of the nose-to-brain delivery.

### *Ex vivo* Permeation Study

**FIGURE 6:**
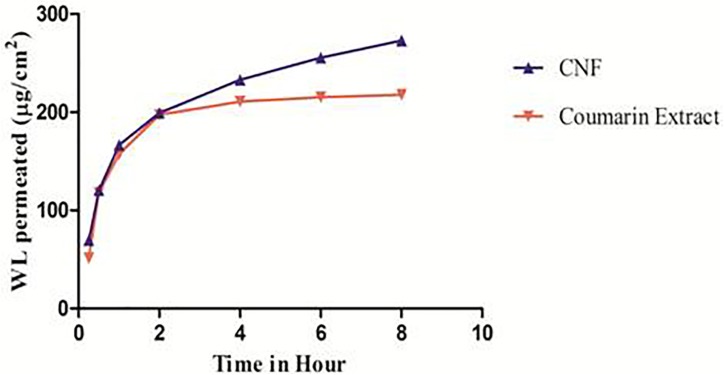
*Ex vivo* permeation pattern of CNF and coumarin fraction. Data presented as mean values ± SD (*n* ≥ 3) by paired *t*-test. Differences were considered statistically significant at *p* < 0.05.

**Figure 6** represents *ex vivo* permeation profile of coumarin fraction and CNF across goat nasal mucosa. This is the first time we are reporting the permeability studies for wedelolactone release from fraction and a particular formulation. The overall enhancement ratio of CNF over coumarin fraction solution was found to be 2.29. At the start, CNF permeates faster than coumarin fraction. It showed 1.36 times faster permeation compared to the coumarin fraction. CNF resulted in a twofold higher permeation (*t*-test, *p* < 0.05) of wedelolactone compared to coumarin fraction solution over a period of 8 h.

### Evaluation of CNF Irritancy Potential on HET-CAM

Prior to the irritation scoring, two different concentrations of the coumarin extract (i.e., 1 and 5%) were subjected to irritation severity scoring. The mean irritation severity score for 1% coumarin extract in CNF was found to be 0.07 and that of 5% was 0.57. So, 1% coumarin extract was used further to formulate CNF.

**FIGURE 7:**
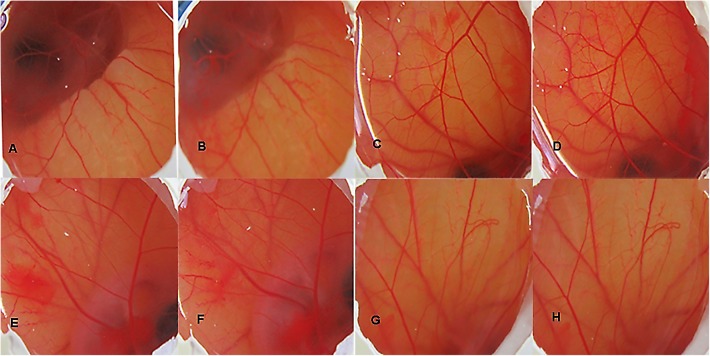
HET CAM bioassay, images of CAM before and after 5 min of contact with the different formulations, DSLR camera images x20: **(A)** Group I (30 s); **(B)** Group I (5 min); **(C)** Group II (30 s); **(D)** Group II (5 min); **(E)** Group III (30 s); **(F)** Group III (5 min); **(G)** Group IV (30 s); **(H)** Group IV (5 min).

The irritancy score as evident from **Table 3** of the negative control group was found to be 11.59 while that of normal saline, vehicle control and CNF was 0. The CNF treated group (i.e., Group IV) as well as vehicle control did not exhibit any irritancy (**Figure 7**). Thus, the mean irritation severity score was zero.

**Table 3:** Irritancy score on HET-CAM for given formulations.

Groups/image no	HT	LT	CT	Irritancy score
Group 1 (normal)	300	300	300	0.07
Group 2 (vehicle control)	300	300	300	0.07
Group 3 (negative control)	7.67	15	300	11.59
Group 4 (CNF)	300	300	300	0.07


*Results are the mean values of *n* = 6.*

### Screening of Coumarin Fraction in Acute Model of PTZ-Induced Seizures in Mice

Coumarin fraction treatment at the dose of 100 mg/kg i.p. dose (*P* < 0.001) and diazepam 2 mg/kg i.p. Dose produced a significant (*P* < 0.001) increase in onset of myoclonic seizure and clonic seizure in the PTZ induced seizures in mice (**Table 4**). Coumarin fraction significantly delayed the onset of HLE and time taken for death (*p* < 0.05 and *P* < 0.001) at 75 mg/kg. Coumarin fraction at 100 mg/kg treatment showed 100% protection from death.

**Table 4:** Screening of coumarins in PTZ induced seizure model in mice. **(A)** Onset of myoclonic jerks. **(B)** Onset of clonic seizures. **(C)** Onset of hind limb extensor (HLE). **(D)** Onset of death.

Treatments	Time in seconds				
	
	Onset of myoclonic jerks	Onset of clonic seizures	Onset of HLE	Onset of death	% Protection from death
PTZ control (100 mg/kg)	49.5 ± 6.69	71.83 ± 7.23	411.33 ± 12.96	411.33 ± 12.96	0
Diazepam 2 mg/kg + PTZ (100 mg/kg)	0 ± 0.00^∗∗∗^	0 ± 0.00^∗∗∗^	0 ± 0.00^∗∗∗^	0 ± 0.00^∗∗∗^	100^∗∗∗^
Coumarin 50 mg/kg + PTZ (100 mg/kg)	49.17 ± 2.15	72 ± 3.3	573.40 ± 89.15	613 ± 105.42^∗^	83.33^∗∗∗^
Coumarin 75 mg/kg + PTZ (100 mg/kg)	57.33 ± 3.56^∗∗∗^	74.5 ± 3.97	686.25 ± 137.94^∗^	820 ± 104.13^∗∗∗^	50.00
Coumarin 100 mg/kg + PTZ (100 mg/kg)	61.83 ± 5.96	104.66 ± 17.29^∗^	0 ± 0.00^∗∗∗^	0 ± 0.00^∗∗∗^	100^∗∗∗^


*Data are expressed as mean ± SEM (*N* = 6). ^∗^*p* < 0.05, ^∗∗^*p* < 0.01, ^∗∗∗^*p* < 0.001 as compared with the PTZ control group; one-way ANOVA followed by Dunnett test.*

The treatment with coumarin fraction (100 mg/kg) and diazepam (2 mg/kg) produced a significant (*P* < 0.001) increase in onset of myoclonic and clonic seizures (**Table 4**). Coumarin fraction significantly delayed the onset of HLE and time taken for death at 75 mg/kg (p < 0.05 and *P* < 0.001). Coumarin fraction at 100 mg/kg treatment showed 100% protection from death.

### Evaluation of Pharmacokinetic and Brain Distribution Parameter of CNF

**FIGURE 8:**
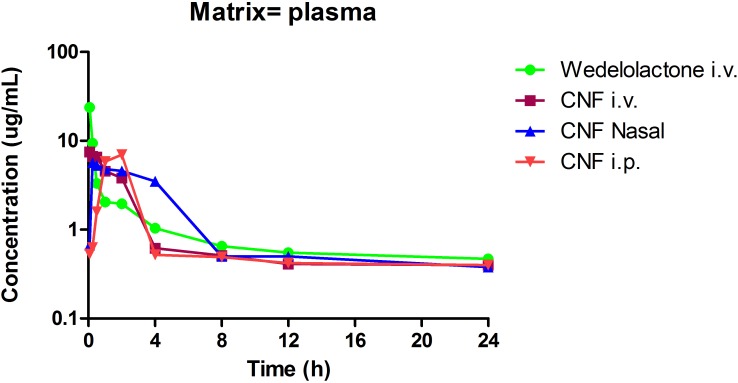
Pharmacokinetic and brain distribution parameter of wedelolactone and CNF.

Based on the encouraging results of the *in vitro* data, pharmacokinetic study was designed further to verify the brain to plasma distribution potential of CNF in rats. The intravenous plasma concentration-time profiles of wedelolactone, CNF (i.v.), CNF (nasal), and CNF (i.p.) (0.4 mg/kg) in rats are shown in **Figure 8**. Brain levels of wedelolactone with CNF (nasal) displayed a 2.43- and 4.01-fold increase in the area under curve as compared to CNF (i.v) and CNF (i.p.), respectively. There was no significant alteration found in pharmacokinetic parameter in plasma matrix of CNF (i.v.), CNF (nasal), and CNF (i.p.) as compared to wedelolactone (i.v.) data. As shown in **Tables 5**, **6**.

**Table 5:** Pharmacokinetic parameter of wedelolactone and CNF.

Formulation	Route	Tmax (h)	Cmax (μg/ml)	C0 (μg/ml)	AUClast (h^∗^μg/ml)	AUC in (h^∗^μg/ml)	Cl (ml/min/kg)	Vss (l/kg)	T1/2 (h)	Clast (μg/ml)	Tlast (h)
CNF	IP	2.00	6.99		24.93	28.01			8.51	0.40	24.00
CNF	IV	0.08	7.54	7.99	23.88	27.71	0.24	0.14	6.64	0.40	24.00
CNF	Nasal	0.25	5.67		32.46	34.76			5.89	0.38	24.00
Wedelolactone	IV	0.08	23.84	37.82	25.18	31.11	0.21	0.16	10.84	0.47	24.00


**Table 6:** Brain distribution parameters of wedelolactone and CNF.

Formulations	Brain (AUC)		Plasma (AUC)		Brain/plasma ratio (AUC)	
			
	12 h	24 h	12 h	24 h	12 h	24 h
Wedelolactone i.v.	3863.6	977.625	1153	635	3	2
CNF i.v.	1192.125	878.15	237	204	5	4
CNF Nasal	2908.325	380.4	803	67	4	6
CNF i.p.	724.775	791.075	283	177	3	4


### Screening of CNF in PTZ-Induced Kindling Model in Mice

**FIGURE 9:**
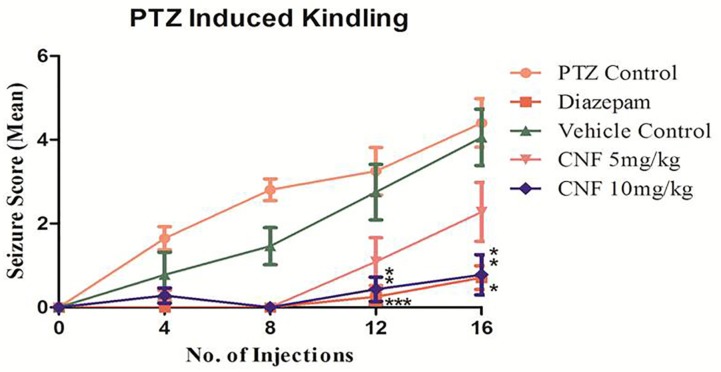
Screening of CNF on seizure score in PTZ kindling model. *N* = 8, data are expressed as mean ± SEM, statistical analysis by one-way ANOVA followed by Dunnett’s test. Significance at *^∗^P* < 0.05, ^∗∗^*P* < 0.01, ^∗∗∗^*P* < 0.001, and ns, not significant vs. PTZ control.

Repeated administration of sub-convulsant dose of PTZ (35 mg/kg) on every alternate day produced kindling in negative control (vehicle + PTZ) group which required 14 injections (30 days) resulting in increased convulsive activity that can be termed as epileptogenesis, leading to generalized clonic–tonic seizure. Diazepam 1 mg/kg i.p. and CNF 10 mg/kg nasal dose showed similar protection, as none of the animals exhibited seizure score of 4–5 during the kindling period. CNF at dose 10 mg/kg nasal dose significantly (*P* < 0.001) reduced both the incidence as well as severity of seizures occurring during the repeated treatments with PTZ during the course of kindling period (**Figure 9**). CNF at 5 mg/kg nasal dose did not protect the animals from seizures and there was no significant reduction in the seizure score. However, the lower dose (5 mg/kg) protected from mortality as well as prevented development of kindling (No 4–5 seizure score).

#### Screening of CNF on Cellular Antioxidants Namely SOD, CAT, GSH, and LPO Levels in PTZ Kindling Model

**FIGURE 10:**
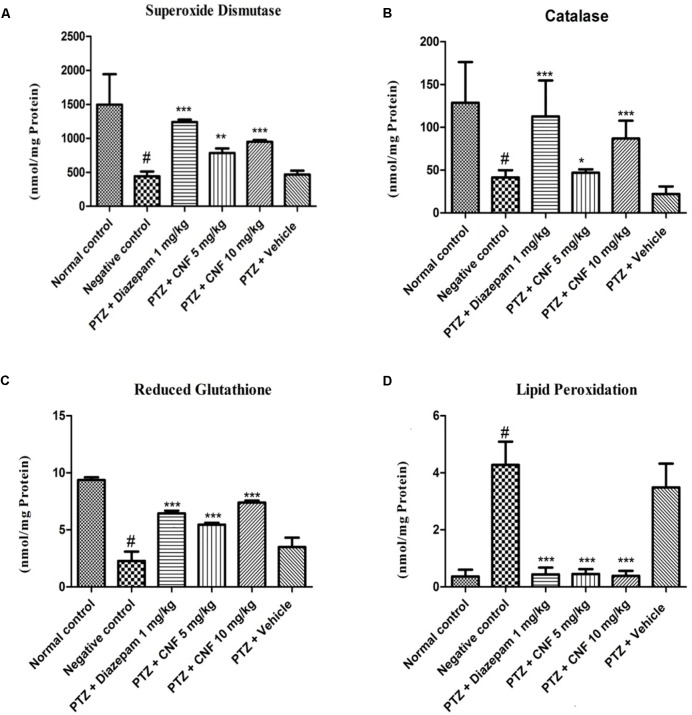
Effect of CNF on **(A)** SOD activity (Fisher et al., 2005), **(B)** CAT activity, and **(C)** GSH and **(D)** LPO levels in midbrain ofPTZ induced kindling model in mice **(A)** SOD (Fisher et al., 2005), **(B)** catalase, **(C)** GSH, and **(D)** LPO. Data are expressed as mean ± SEM (*N* = 6). ^∗^*p* < 0.05, ^∗∗^*p* < 0.01,^∗∗∗^*p* < 0.001 as compared with the PTZ control group and ^#^*p* < 0.05 compared with normal group; one-way ANOVA followed by Dunnett test.

Pentylenetetrazole induced kindling caused a remarkable decrease in the activities of the major antioxidant enzymes, i.e., SOD and CAT (*P* < 0.001, *P* < 0.05). CNF at 5 and 10 mg/kg significantly increased the activity of SOD and CAT (*P* < 0.05, *P* < 0.01) whereas at vehicle control did not exhibit any effect on these antioxidant enzymes. Moreover, diazepam significantly increased the SOD and CAT activity (*P* < 0.01, *P* < 0.05; **Figure 10**). GSH level in PTZ control group was significantly (*p* < 0.001) lower than the normal control group. CNF at 5, 10 mg/kg (*P* < 0.01, *P* < 0.001) and diazepam (*P* < 0.01) showed significant increase in the levels of GSH as compared to PTZ control. The lipid peroxidation (MDA level) was markedly increased in the negative control group which was significantly (*P* < 0.001) higher than that seen in the vehicle control group. CNF and diazepam (*P* < 0.01) treated groups showed significant reductions in the levels of MDA as compared to negative control group.

#### Histopathology

**FIGURE 11:**
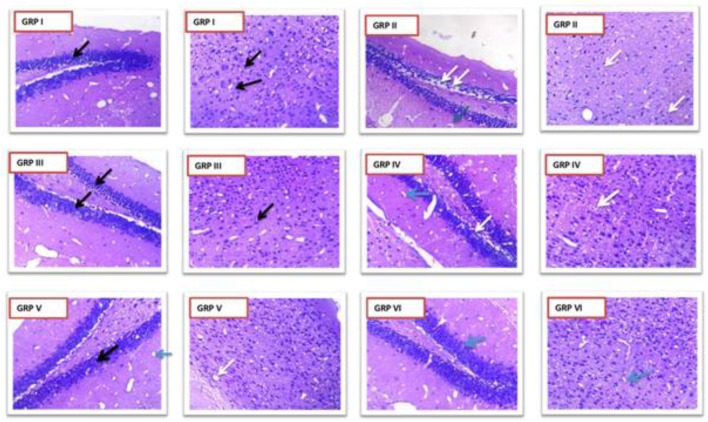
Photomicrographs showing changes in histopathology of neuron. GRP-I standard control (PTZ + diazepam), GRP-II negative control (PTZ), GRP-III normal control, GRP-IV PTZ + vehicle control, GRP-V PTZ + CNF 5 mg/kg, GRP VI- PTZ + CNF 10 mg/kg Black arrows: intact neurons with basophilic cytoplasm and prominent nucleus. White arrows: degenerative neurons with pyknotic nucleus. Blue arrows: Hirani bodies.

Hematoxylin and eosin staining as per Fujikawa scaling system were applied. Group II showed dead neurons with pyknotic nuclei [Score 2.5 (55–75% damage)] which were clearly distinguishable from surviving cells that showed round-shaped, cytoplasmic membrane-intact cells, without any nuclear condensation or distorted aspect (**Figure 11**). Group I (standard) V and VI (treatment groups) (Score 0 – no damage) improved the seizure induced neuronal damage in the treatment groups in kindled mice. Coumarin at 10 mg/kg [Score 1.75 (25–45% damage)] did not protect from seizure mediated neuronal damage. Moreover, diazepam 1 mg/kg prevented the neuronal damage as a result of kindling.

#### Immunohistochemistry

**FIGURE 12:**
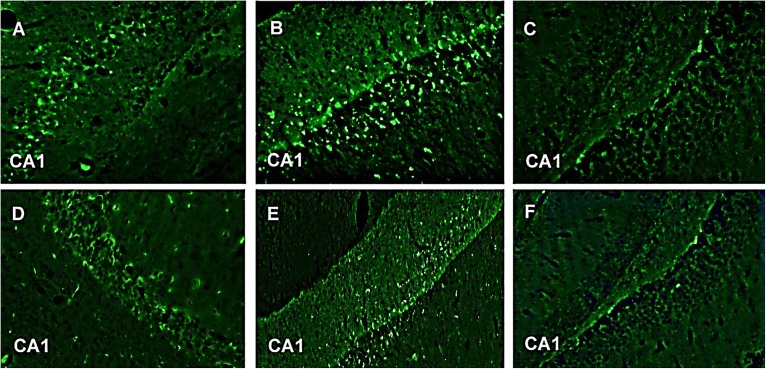
GFAP-FITC immunohistochemistry. Representive photomicrographs of GFAP-immunoreactive neurons in CA1 region of mice hippocampus. **(A)** PTZ + diazepam, **(B)** PTZ, **(C)** normal, **(D)** PTZ + vehicle, **(E)** PTZ + CNF 5 mg/kg, and **(F)** PTZ + CNF 10 mg/kg.

In our study, GFAP-fluorescein (FITC) exhibited fluorescent astroglial cells particularly in CA1 region of hippocampus. This is the first time we are reporting immunohistochemistry where astroglial cells show fluorescence after taking GFAP-FITC. Astroglial cells produce GFAP during neuronal injury. These damaged neurons contain GFAP which when bound to anti-GFAP antibody (which in turn is bound to the secondary antibody alexafluor 488) gives fluorescence. Group III (normal control) and Group VI (CNF 10 mg/kg) did not show any fluorescence indicating the absence of GFAP in the astroglial cells of CA1 region of hippocampus. Group V (CNF 5 mg/kg) and Group I (diazepam 1 mg/kg) showed little fluorescence with no distortion in the morphologies of the neurons in CA1 region but, on the other hand, Group II (PTZ-controlled) and Group IV (vehicle-controlled) showed strong fluorescence indicative of inflammations in the damaged neurons of CA1 region (**Figure 12**). Thus, Group II and Group IV showed significantly higher levels of GFAP (*P* < 0.001) compared to the other groups.

#### Determination of TNF α by ELISA

**FIGURE 13:**
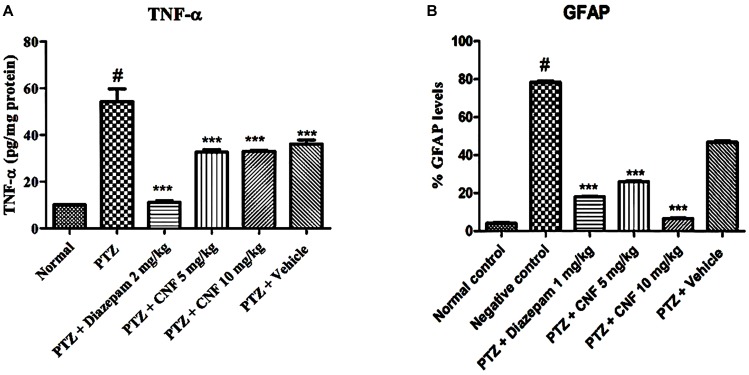
Effect of CNF onTNF-α **(A)** and GFAP **(B)** in hippocampus. Data expressed as mean ± SEM. N-6. ^∗∗∗^*P* < 0.001 compared with PTZ group and #*P* < 0.01, compared with normal group using one-way ANOVA followed by Dunnett’s test as a post-ANOVA test.

TNF-α, a pro-inflammatory marker, forms during injury and is estimated using an ELISA technique in the brain homogenate. As depicted in **Figure 13**, PTZ causes significant elevation of level of TNF-α in negative control group (*P* < 0.001) as compared to the normal control group. Treatment with CNF 5, 10 mg/kg (*P* < 0.001) and diazepam (*P* < 0.001) significantly attenuated the levels of TNF-α in comparison to the negative controlled group.

## Discussion

Epilepsy is a complex neurological disorder which has plagued mankind since ages and continues to afflict more than 50 million people across the globe. It is a spectrum disorder characterized by recurrent seizures due to abnormal excessive and synchronous neuronal activity in the brain. The current medications primarily act to symptomatically suppress seizures rather than correcting the underlying abnormalities causing epilepsy or altering its natural histopathology. Furthermore, trepidations about the safety (narrow therapeutic window) and costs of the available drugs is a major concern. There are no direct reports in literature indicating the anticonvulsant effect of coumarin fraction of EA, however the activity of methanolic extract of EA in PTZ and MES models is reported. The study was undertaken to investigate the neuroprotective activity of CNF and to identify the active principle responsible for the antiepileptic activity. Primarily coumarins isolated from the crude extract of EA were screened in the acute PTZ induced seizure model in mice. This was on the basis of previous study carried by (Shaikh et al., 2012) at ICT, which confirmed the antiepileptic activity of crude extract of EA. FTIR and HPLC analysis of CNF confirms the presence of wedelolactone, luteolin, and apigenin in the coumarin fraction. *In vitro* analysis and *ex vivo* permeation results confirmed the immediate release, enhanced permeation, and nose to brain delivery of CNF for its possible effect (Westin et al., 2006).

Coumarin fraction (100 mg/kg) exhibited an excellent anticonvulsant activity in PTZ test. PTZ induced seizure model is preliminary screening model to assess the anticonvulsant potential of a substance as it gives predictive relevance regarding the clinical spectrum of the investigational compound (Jain et al., 2015). PTZ test is assumed to identify drugs effective against human generalized absence and tonic–clonic seizures (Sayyah et al., 2005). To further confirm the anticonvulsant activity of coumarins and on the basis of previous results; CNF was formulated using coumarin fraction from methanolic extract of EA. It was further evaluated and studied in PTZ induced kindling model of mice. Kindling is a very widely accepted and a suitable model for studying the process of epileptogenesis. Kindling is also a model of brain plasticity in which recurrent activation of neural pathways results in an increased vulnerability to evoked seizures that ultimately progresses to impulsive seizures (de Almeida Rabello Oliveira et al., 2008). This model is characterized by an amplified susceptibility to seizures after recurring brain stimulation with subcunvulsive stimuli. It has been shown that an effect comparable to electric kindling can be induced by the repeated administration of subconvulsant doses of central nervous system stimulants (Corda et al., 1991). Thus, a progressive development of seizures, i.e., chemical kindling, is observed after the repetitive (on every alternate day) administration of PTZ (35 mg/kg; Tambe et al., 2016). Data from this study showed that the convulsion produced by PTZ was significantly delayed by CNF. In the various kindling experiments carried out, subconvulsive dose of PTZ (35 mg/kg) when given *i.p.* on alternate days induced kindling in PTZ treated mice on 14th injection of PTZ. During the course of kindling, following the subconvulsive doses of PTZ, there is a gradual increase in susceptibility to seizures ultimately leading to generalized tonic–clonic seizures. Administration of CNF (5 and 10 mg/kg) significantly decreased the seizure score as compared to PTZ treated animals which produced maximum seizure score. It was also observed that administration of CNF (5 and 10 mg/kg) did not produce kindling till 24 PTZ injections similar to diazepam 1 mg/kg i.p. (in a different set of animals, data not presented). This indicated increased latency to seizure onset implying neuroprotection offered by the formulation when administered by intranasal route. Oxidative stress causes an imbalance which leads to higher cellular building of reactive oxygen species (ROS) like superoxide radical (O_2_^-^, hydrogen peroxide (H_2_O_2_) etc.). This built up of reactive radicals reduces cellular antioxidant defense (SOD, CAT, and reduced GSH). Brain tissue is highly susceptible to the oxidative damage due to its high demand and consumption of oxygen. Seizures cause alterations in the membrane lipid composition, perturbing the membrane fluidity and permeability resulting in the disturbances in functioning of membrane bound enzymes which have deleterious consequences on neuronal functioning (Corda et al., 1992). In the current studies, PTZ induced kindling significantly decreased the ROS scavenging activity of normal cellular antioxidants, namely, SOD, CAT, and GSH in the brain which was in line with Ginkgo biloba extract (Ilhan et al., 2006). CNF (5 and 10 mg/kg) ameliorated the SOD and CAT enzyme activities and restored the levels of GSH. The increase in levels of MDA (an end product of free radical generation) and simultaneous decrease in the levels of GSH (free radical scavenger) in vehicle-PTZ treated mice indicated free radical generation on administration of subconvulsive dose of PTZ. The treatments, CNF 5, and 10 mg/kg caused a significant decrease in MDA levels in comparison to PTZ treated mice offering protection to neuronal cells. This might be attributed to the free radical scavenging potential consequently attenuating seizures (Bikjdaouene et al., 2003). The data also indicated that diazepam antagonized PTZ convulsion. PTZ may be exerting its convulsant effect by inhibiting the activity of gamma amino butyric acid (GABA) at GABA_A_ receptors. GABA is the major inhibitory neurotransmitter implicated in epilepsy (De Sarro et al., 1999). The enhancement and inhibition of the neurotransmission of GABA will attenuate and enhance convulsions, respectively (Meldrum, 1981; Gale, 1992). Diazepam, a standard AED, has been shown to exert its antiepileptic effects by enhancing GABA-mediated inhibition in the brain (Porter and Meldrum, 2001). It is possible that diazepam antagonized PTZ convulsion in this study by enhancing GABA neurotransmission (Amabeoku et al., 2007). PTZ is reported to interact with the GABA neurotransmission and the GABA receptor complex antagonism by PTZ leads to PTZ-induced seizures (Bum et al., 2001). Since CNF was found to be active against PTZ convulsions, it is probable that it may be interfering with GABA mechanism by enhancing the activation of GABA_A_ receptors owing to the phytoconstituents present in it (Kundaikar et al., 2015). This would facilitate the GABA-mediated opening of chloride channels offering symptomatic relief. HPLC results confirmed presence of wedelolactone, a naturally occurring coumarin as major phytoconstituent in coumarin fraction and CNF. It also confirmed the presence of flavonoids, namely, luteolin and apigenin in the coumarin fraction. Reports suggest that wedelolactone has selectivity and affinity toward benodiazepine binding site which is an allosteric site on GABA receptor (Po^∧^ças et al., 2006).

Astrogliosis is characterized by the hypertrophy of the cell bodies and processes of the astrocytes (a major neuralglia) along with increased expression of GFAP in response to various threats (Sherafat et al., 2013). Astrogliosis is an important feature of the epileptic foci and presence of GFAP is considered as a marker of neuroinflammation. The transformation of astrocytes to reactive astrocytesin epileptic disorder (especially in the kindling models) could be associated with the adaptive changes during disease progression and most likely precedes neuronal damage (Franke and Kittner, 2001). Series of repeated seizures in the kindled animals in the present study, shows increased GFAP immunoreactivity. The kindled animals treated with CNF showed significantly lower GFAP levels, which may be due to the anti-inflammatory activity of wedelolactone through inhibition of LPS-induced inflammation via NF-kappa B pathway (Yuan et al., 2013).

## Conclusion

In conclusion, the report exemplifies the neuroprotective antiepileptogenic effect of CNF along with its anticonvulsant effect. In the present study, coumarin fraction was formulated for nasal delivery and evaluation of antiepileptic activity of the same.

The safety and non-irritant potential of CNF as a nasal delivery was confirmed in *in vitro* HET-CAM analysis. *Ex vivo* permeation data demonstrated considerable enhanced permeation of wedelolactone across the goat nasal mucosa from CNF compared to the coumarin fraction. *In vivo* experimental findings substantiate the neuro-protective, anti-oxidant, anti-inflammatory, and disease modifying effects of CNF in mitigating the sequel of events implicated in the progression of epileptic disorders/seizures. This study rationalizes CNF as a promising newer formulation in forestalling the process of epileptogenesis and associated co-morbidities.

## Author Contributions

SM performed extraction, formulation, development, and characterization. SM, VP, AK, and SB performed animal studies, biochemical, histological, and TNF-α analysis and analyzed the data. SM and VD performed the immunohistochemistry. SS designed the research, formulation, and supervised the project. SM, AK, and SS contributed to writing and editing of the manuscript.

## Conflict of Interest Statement

The authors declare that the research was conducted in the absence of any commercial or financial relationships that could be construed as a potential conflict of interest. The handling Editor declared a past collaboration with one of the authors SS.

## Abbreviations

AED: antiepileptic drug
ANOVA: analysis of variance
BBB: blood–brain barrier
CA1: Region 1 Cornu Ammonis
CAT: catalase
CNF: coumarin nasal formulation
CT: coagulation time
EA: *Eclipta alba*
FITC: fluorescein
FTIR: Fourier transform-infrared
GABA_A_: gamma-aminobutyric acid (GABA) type A receptor
GFAP: glial fibrillary acidic protein
GRP: group
GSH: glutathione
H&E: hematoxylin and eosin
HET-CAM: hen’s egg test choroiallantoic membrane
HPLC: high performance liquid chromatography
HT: hemorrhage time
ICH: International Council for Harmonization of Technical Requirement
i.p.: intraperitoneal
i.v.: intravenous
LT: lysis time
MDA: malondialdehyde
MES: maximum electroshock
NFκB: nuclear factor kappa-light-chain-enhancer of activated B-cells
PBS: phosphate buffer saline
PDA: photo diode array
PTZ: pentylenetetrazole
SD: standard deviation
SEM: standard error of mean
SNES: simulated nasal electrolyte solution
SOD: superoxide dismutase
TNF-α: tumor necrosis factor-alpha
UV: ultraviolet
WL: wedelolactone
